# Resilience and sense of agency among healthcare professionals during crisis

**DOI:** 10.3389/fpubh.2026.1790636

**Published:** 2026-03-04

**Authors:** Yonatan Link, Christopher Groombridge, Moran Bodas, Nir Samuel

**Affiliations:** 1 The School of Public Health, Gray Faculty of Medical and Health Sciences, Tel-Aviv University, Tel-Aviv-Yafo, Israel; 2 National Trauma Research Institute, Melbourne, VIC, Australia; 3 Emergency and Trauma Centre, The Alfred Hospital, Melbourne, VIC, Australia; 4 School of Translational Medicine, Monash University, Melbourne, VIC, Australia; 5 The Pediatric Trauma Service & The Department of Emergency Medicine, Schneider Children's Medical Centre of Israel, Petah Tikva, Israel

**Keywords:** agency, health care professionals, Mass casualty event (MCE), psychological capital, resilience

## Abstract

**Background and objectives:**

Healthcare professionals (HCPs) are central to emergency response, and their effectiveness depends not only on clinical expertise but also on psychological resources. Strengthening these resources may sustain workforce capacity and safeguard care under crisis conditions. This study examined the contribution of psychological capital—particularly resilience—to perceived agency among Israeli HCPs during mass casualty incidents and violent conflict after 7th of October 2023. The objective was to assess whether psychological resources predict perceived effectiveness beyond traditional factors, with implications for preparedness, organisational support, and policy. Findings may inform interventions relevant to resource-restricted environments and adaptable to developing countries.

**Methods:**

The study was designed as an observational-cross-sectional study, conducted using encrypted online questionnaires distributed via QR codes in trauma centres, dispatch stations, and professional forums. Participants were qualified physicians, surgeons, nurses, paramedics, and EMTs providing patient care during the conflict. Instruments measured psychological capital (PCQ-24), resilience (CD-RISC 10), and perceived agency (RSES).

**Results:**

204 HCPs participated (mean age 37.4, SD = 11.6; 61.8% male). Psychological capital (*r* = 0.387, *p* < 0.001) and resilience (*r* = 0.485, *p* < 0.001) were strongly associated with agency, surpassing prior experience. Paramedics/EMTs reported higher agency than nurses/physicians (*p* < 0.001). Spirituality also predicted agency (*β* = 0.142, *p* = 0.022).

**Conclusion:**

Psychological capital and resilience are key determinants of HCPs’ perceived agency in crises. Embedding resilience-building into public health systems, alongside organisational and policy measures, may enhance workforce readiness across conflict, disaster, and health threats. These findings are especially relevant to resource-limited settings, where psychological preparedness may be a cost-effective means to support frontline staff and sustain delivery.

## 1 Introduction

Emergencies, whether natural or man-made, are unforeseen and characterized by elements of uncertainty, and significant harm to individuals, communities, and the environment (1). In such crises, healthcare professionals (HCPs) encounter multifaceted challenges, including self-imposed and societal expectations to sustain optimal performance under substantial mental and cognitive strain (2, 3). Psychological capital has been recognized as a key factor in mitigating the mental effects of crisis (4).

Psychological capital (PsyCap) is a construct comprising self-efficacy, hope, optimism, and resilience, which plays a crucial role in disaster scenarios. Studies have shown that PsyCap is positively correlated with disaster preparedness and coping styles (5). For HCPs involved in disaster response, higher PsyCap levels are associated with lower secondary traumatic stress (6). PsyCap is significant in enhancing individual and organizational resilience, reducing psychological distress, and improving disaster preparedness across various sectors affected by catastrophic events (7, 8).

Resilience is a pivotal part of PsyCap and is conceptualized as the capacity to avoid succumbing to distress, function at or near pre-crisis levels and later “bounce back” or even “bounce forward,” achieving post-traumatic growth (9). For individuals assuming key roles in emergency scenarios, resilience is an essential resource to cope and perform under adversity (2, 10). Agency refers to the ability of an individual to act effectively in a given environment. Subjectively, it reflects an individual’s perception of self-efficacy, mainly being able to purposely initiate and successfully execute actions and a sense of ownership over their consequences. A positive sense of agency has been linked to successful functioning. A lack agency has been linked to adverse outcomes and post-traumatic stress disorder (PTSD) (11).

On October 7th, 2023, Israel experienced the country’s largest ever mass casualty event (MCE), one of the largest civilian MCEs in modern history. The day ended with 1,200 fatalities, 1,455 surviving casualties, and 239 hostages taken (12, 13). Medical teams were mobilized to address these overwhelming challenges, operating under extreme and prolonged stress. Many medical teams were impacted directly with numerous providers injured or killed. Providers were required to perform while managing the uncertainty for the fate of loved ones, and at times receiving devastating news. The healthcare system was challenged with an unprecedented surge of patients and at times scarcity of resources on the national, organizational and provider levels. October 7th, and the tragic events that ensued presented extreme societal, organizational and personal challenges.

While existing literature has examined psychological capital and resilience in healthcare settings separately (14, 15), and several studies have explored the mental health consequences of the October 7th attacks on the general population (16, 17), significant gaps remain. First, most studies focus on either organizational or individual psychological factors in isolation, rather than examining their interplay during sustained crisis conditions. Second, research on HCPs’ perceived agency, a critical determinant of performance and psychological outcomes, during prolonged mass casualty events, remains limited. Third, cross-professional comparisons (physicians, nurses, paramedics, emergency medical technicians (EMTs)) across different treatment environments (pre-hospital vs. hospital) during extended conflict are scarce. This study addresses these gaps by examining the contribution of psychological capital and resilience to perceived agency among diverse HCPs who provided care during an unprecedented and sustained crisis. The novelty of our research lies in its comprehensive approach as a cross-disciplinary medical sample, spanning multiple professional roles and settings. In addition, the research examines an ongoing, prolonged conflict rather than a discrete event, and focuses on perceived agency as an outcome, which has implications for both immediate performance and long-term psychological wellbeing.

## 2 Methods

### 2.1 Study design

This study was designed as an observational, cross-sectional investigation conducted among Israeli healthcare professionals (physicians, surgeons, nurses, paramedics, and EMTs) who actively provided medical care in both pre-hospital and hospital settings during the ongoing conflict in Israel. The estimand of interest was the population-level association between resilience and perceived agency among healthcare professionals during the defined conflict period. The study setting encompassed pre-hospital emergency medical services and hospital-based care across Israel. For the purposes of this study, the “current conflict” was operationalized as the period from October 7th, 2023, marking the mass casualty event that initiated the regional conflict in Israel, through June 11th, 2024 (the end of data collection). This period was characterized by sustained operational demands on the healthcare system, including ongoing casualty care, heightened security threats, and prolonged organizational strain.

### 2.2 Study population

The study population consisted of Israeli medical professionals who actively provided care between October 7th, 2023, and June 11th, 2024 (from crisis beginning to study data collection commencement). Eligible participants included emergency medical technicians (EMTs), paramedics, nurses, physicians, and surgeons. Inclusion criteria required participants to possess an official medical qualification and actively engage in patient care. Individuals <18 years old, those lacking certification, and HCPs who did not actively provide patient care were excluded.

**Figure 1:**
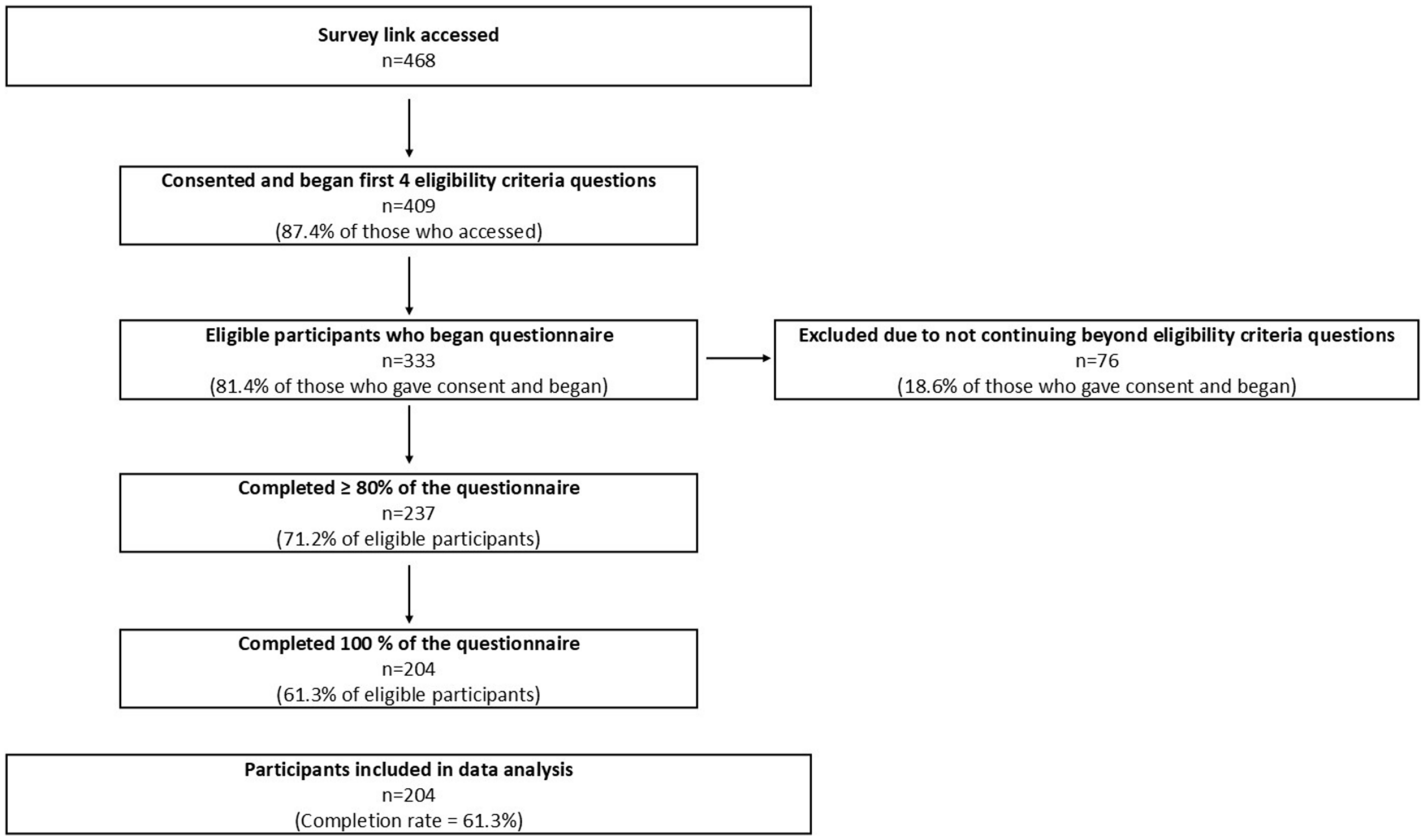
Study population - participant flow diagram. Eligibility criteria required participants to be ≥ 18 years of age, official medical qualification (EMT, paramedic, nurse, physician, or surgeon), and to have actively provided patient care during the conflict period from October 7th, 2023, through June 11th, 2024. The survey was distributed via QR codes in trauma centres, emergency dispatch stations, and electronically through professional forums and social media using snowball sampling (this limits the ability to present the amount of people approached). Completion rate among eligible participants - 61.3%.

The questionnaire link was accessed by 468 individuals. Of these, 59 did not proceed beyond the consent page. Among the remaining 409 individuals who provided consent, 333 (81.4%) met the eligibility criteria and initiated the questionnaire. Of the eligible participants, 237 (71.2%) completed at least 80% of the survey items, and 204 (61.3%) completed the questionnaire in full. The final analytic sample therefore comprised 204 healthcare professionals who provided complete responses to all study instruments. The overall completion rate was 61.3% among eligible participants who initiated the questionnaire. The detailed participant flow is presented in Figure 1.

### 2.3 Variables and tools

#### 2.3.1 Demographic data and professional characteristics

Participants provided data on socio-demographic characteristics, professional background, and personal exposure to threats.

#### 2.3.2 Past exposure and stress characteristics

Past exposure and stress characteristics were assessed using items adapted from the conceptual framework established by Fergusson et al.’s (14) study of the Canterbury (New Zealand) earthquake sequence. That study demonstrated that disaster exposure could be reliably measured (*α* = 0.92) by assessing two domains - immediate impacts of the earthquakes and consequential ongoing disruptions following the disaster. This approach significantly predicted mental health outcomes 20–24 months post-disaster, establishing that measuring past exposure to high-stress conditions is both methodologically feasible and clinically meaningful (14).

Following this principle of measuring both exposure and its consequences, we developed items appropriate specifically to healthcare providers during conflict. We assessed - prior professional experience in high-stress scenarios (mass casualty events, natural disasters, military operations, war), direct exposure to threat and harm (proximity of residence to conflict zones, provision of care under threat, personal or colleague injuries, personal losses), and ongoing frequency and intensity of professional and personal stressful events since October 7th. Like the original research, we used binary responses (yes/no) for categorical exposure variables and multi-point scales (7-point Likert: 1 = infrequent, 7 = very frequent) for frequency and intensity measures.

#### 2.3.3 Measures of psychological capital and resilience

Psychological capital was measured using the Psychological Capital Questionnaire (PCQ-24), developed by Luthans, Avolio, and Avey in 2007 (15). The PCQ-24 provides both overall and subscale scores for four dimensions- hope, efficacy, resilience, and optimism. Scores range from 1 to 6, representing the average response to items within each category on a 6-point Likert scale (1–6), with higher scores reflecting greater psychological resources. The PCQ-24 has demonstrated strong reliability in prior studies with an internal consistency of *α* = 0.92 (16).

Resilience, a key component of psychological capital, was further assessed using the 10-item Connor-Davidson Resilience Scale (CD-RISC 10), derived from the original CD-RISC 25 questionnaire developed by Connor and Davidson in 2003 (17, 18). This scale generates scores ranging from 0 to 40, calculated as the sum of responses to all items on a 5-point Likert scale (0–4), with higher scores indicating greater resilience. The reliability of the CD-RISC 10 has been validated in previous research with an internal consistency of *α* = 0.91 (19).

While resilience is conceptually nested within PsyCap as one of its four dimensions, we measured it separately for two main reasons. First, resilience has been identified as particularly crucial in crisis contexts and warranted deeper examination beyond its representation in the PCQ-24. Second, the use of two validated resilience measures allows for convergent validation and provides a more comprehensive assessment of this critical construct, both as part of the broader PsyCap framework and as a standalone construct.

#### 2.3.4 Measures of perceived agency

Perceived agency, the primary outcome, was evaluated using the Response to Stressful Experiences Scale (RSES), developed by Johnson and colleagues in 2011 (20), The 22-item scale measures individual differences in cognitive, emotional, and behavioral responses to stressful life events, utilizeing a 5-point Likert format, producing scores ranging from 0 to 88, where higher scores indicate greater perceived agency. The RSES was originally developed and the reliability of the RSES has been validated in U. S. military populations, with an internal consistency of *α* = 0.92 (20).

Factor analysis identified five protective factors: meaning-making and restoration, active coping, cognitive flexibility, spirituality, and self-efficacy—all contributing to agency during stressful events. In military and first responder populations, RSES scores have predicted successful psychological adaptation to combat and operational stress, making it particularly appropriate for assessing HCPs facing crisis conditions (20). The scale’s focus on growth-oriented responses to stress, aligns with our conceptualization of perceived agency as encompassing both sense of control and capacity for purposeful action under adversity.

### 2.4 Tool validation

A pilot study with 30 participants was conducted to test the internal validity of the translated instruments, which underwent a standard forward–backward translation and linguistic validation. Each measure was required to achieve Cronbach’s *α* > 0.7 and to demonstrate clarity and alignment with the study aims. All instruments exceeded thresholds (CD-RISC 10: α = 0.84; PCQ-24: α = 0.82; perceived agency: α = 0.88). Participant feedback was used to refine the final questionnaire.

### 2.5 Study procedure

Data were collected via an encrypted web-based platform (Qualtrics) between May 30th and June 11th, 2024, using convenience and snowball sampling. To enhance accessibility across diverse healthcare professionals (HCPs), the questionnaire was distributed in both physical and online settings. QR codes were placed in four Level I trauma centres in central Israel and Jerusalem, urgent care clinics, and three regional emergency medical dispatch centres. The questionnaire link was also shared through professional forums and social media platforms. Snowball sampling was used to encourage participants to invite colleagues.

Participation was voluntary and anonymous, and no incentives were provided. Due to the anonymous design and snowball sampling methodology, specific institutional affiliations were not systematically recorded. This approach enabled broader geographic and institutional representation while preserving participant confidentiality. Respondents represented a diverse range of healthcare settings across Israel, including Level I trauma centres, community hospitals, and pre-hospital emergency services operating in both civilian and military contexts.

### 2.6 Statistical analysis

Based on prior studies (21) and accepted standards, a minimum of 163 participants was calculated to ensure 80% power to detect an effect size of 0.8 with 95% CIs. Analyses were performed using IBM SPSS® v29.0. Continuous variables were summarized as means ± SD and categorical variables as frequencies/percentages. Normality was tested with Kolmogorov–Smirnov; parametric tests were applied given sample size. Univariate analyses included t-tests, Pearson correlations, and ANOVA with Bonferroni corrections. Multicollinearity and homoscedasticity were checked (VIF < 1.91). Multivariate linear regression identified predictors of perceived agency, with significance at *p* < 0.05 (unless adjusted).

## 3 Results

### 3.1 Characteristics of participants

A total of 204 HCPs participated in the study. The mean age was 37.4 (SD = 11.6, range 18–68) years, with 61.8% males. The sample included both civilian (74%) and military (26%) HCPs, across diverse professional backgrounds and experience levels. Detailed demographic and professional characteristics are presented in Table 1.

**Table 1:** Demographic and professional characteristics and associated perceived agency scores (response to Stressful Experiences Scale).

Variable	Total (*n* = 204)	Perceived agency score (M ± SD) ^*^	*p* value
Demographic characteristics			
Age (y), M ± SD (min-max)	37.4 ± 11.6 (18–68)	r = −0.015	0.836^a^
Gender			
Male	126 (61.8%)	64.6 ± 11.0	0.248^b^
Female	77 (37.7%)	66.4 ± 10.2	
Other	1 (0.5%)		
Family status			
Single	57 (27.9%)	63.7 ± 9.4	0.091^b^
In a relationship	147 (72.1%)	66.4 ± 10.8	
Parents of children	130 (63.7%)	66.0 ± 11.0	0.591^b^
Education^d^			
Non-Academic	57 (27.9%)	69.5 ± 10.4	< 0.001^b^
Academic	147 (72.1%)	64.1 ± 10.1	
Religiosity			
Secular	70 (34.3%)	63.0 ± 9.7^**^	0.012^c^
Traditional	34 (16.7%)	67.1 ± 9.5	
Devout	60 (29.4%)	65.3 ± 11.4	
Very devout	40 (19.6%)	69.6 ± 10.2^**^	
Professional characteristics			
Profession			
Physician/Surgeon	58 (28.4%)	62.6 ± 9.8^**^	< 0.001^c^
Nurse	38 (18.6%)	62.3 ± 9.8^**^	
Paramedic/EMT	108 (53.0%)	68.5 ± 10.3^**^	
Years of experience (y), M ± SD (min-max)	10 ± 8.5 (0–41)	r = −0.036	0.613^a^
Treatment environment			
Pre-hospital	77 (37.7%)	69.2 ± 10.5^**^	< 0.001^c^
Military	53 (26.0%)	65.2 ± 10.1	
Acute Care^e^	49 (24.0%)	62.6 ± 9.7^**^	
Other	19 (9.3%)	61.1 ± 10.5^**^	
Provided care in their pre-conflict treatment environment			
Yes	126 (61.8%)	65.0 ± 10.7	0.326
No (Different treatment setting^f^)	75 (36.7%)	66.5 ± 10.1	
Unknown	3 (1.5%)		

^a^Based on Pearson correlation; ^b^Based on independent sample t-test; ^c^Based on One-Way ANOVA; ^d^Non-Academic – high school or vocational. Academic – bachelor’s and above; ^e^Emergency department, ICU and Surgical department; ^f^Deployed or allocated to a different department or setting, e.g., hospital to prehospital, different department or different medical facility. ^*^Perceived agency score is represented as M ± SD for categorial variables and as Pearson correlation for continuous variables. ^**^Multiple comparisons using Bonferroni test – presenting the significant categories.

### 3.2 Psychological capital and resilience of participants

The mean psychological capital score, assessed using the PCQ-24, was 4.7 (SD = 0.6, range 3.1–6.0). Detailed subscale scores are presented in Table 2. The mean resilience score, measured by the CD-RISC-10, was 32.0 (SD = 4.9, range 19–40). Overall, these findings reflect moderate to high levels of psychological capital and resilience within the sample, indicating that healthcare professionals who continued providing care during the ongoing conflict exhibited relatively strong psychological resources. Table 2 further summarizes descriptive statistics for psychological measures.

**Table 2:** Descriptive statistics for resilience and psychological capital scores.

Variable	(M ± SD)	Min-Max	Median	IQR
Resilience Levels (CD-RISC 10 score)	32.0 ± 4.9	19.0–40.0	32.0	28.3–36.0
PCQ 24 – General score	4.7 ± 0.6	3.1–6.0	4.7	4.3–5.1
PCQ 24 – Hope component	4.7 ± 0.7	2.3–6.0	4.7	4.3–5.2
PCQ 24 – Efficacy component	4.6 ± 0.9	2.0–6.0	4.7	4.0–5.3
PCQ 24 – Resilience component	4.9 ± 0.6	3.0–6.0	5.0	4.4–5.4
PCQ 24 – Optimism component	4.6 ± 0.8	1.75–6.0	4.5	4.3–5.0

### 3.3 Perceived agency and associated factors

We applied three tools to assess participants’ psychological capital, resilience and perceived agency: The PCQ-24 for psychological capital, the CD-RISC 10 for resilience and the Response to Stressful Experiences Scale (RSES) for perceived agency.

#### 3.3.1 Demographic characteristics

Professional role exhibited the largest effect size, with paramedics and EMTs reporting higher perceived agency scores compared to doctors and nurses (*F*_(3,200)_ = 6.128, *p* < 0.001). This was followed by the treatment environment, where individuals working in pre-hospital settings reported greater perceived agency than those in hospital-based settings, independent of their roles (e.g., paramedic, nurse, etc.) (*F*_(3,194)_ = 5.847, *p* < 0.001). Regarding education, individuals holding non-academic qualifications reported higher perceived agency scores than academically trained providers (t_(202)_ = 3.368, *p* < 0.001). Religiosity was significantly associated with perceived agency, as participants identifying as highly devout consistently demonstrated higher perceived agency scores (*F*_(3,200)_ = 3.759^1^, *p* = 0.012), suggesting a potential link between spiritual beliefs and resilience under stress. We did not find a significant association between perceived efficacy and the number of years of experience in the role (r = −0.036, *p* = 0.613).

#### 3.3.2 Past exposure

We examined the relationship between various exposure factors and perceived agency. Despite diverse previous exposure to significant stressors, most variables did not show a clear association with perceived agency scores (Table 3). Prior experience in stressful conditions (Table 3) did not yield significant differences in perceived agency (t_(202)_ = 0.457, *p* = 0.649). Direct exposure to danger, such as treating patients under threat (t_(196)_ = −0.088, *p* = 0.930) or experiencing personal/colleague injuries (t_(194)_ = −0.435, *p* = 0.664), showed no significant impact on perceived agency. The frequency of stressful professional events (r = 0.016, *p* = 0.822) and personal events (r = −0.083, *p* = 0.236) showed no significant correlations with perceived agency.

**Table 3:** Exposure characteristics and associated perceived agency score (response to Stressful Experiences Scale).

Variable	Total (*n* = 204)	Perceived agency score (M ± SD) ^*^	*p* value
Previously provided care under stressful conditions			
No	81 (39.7%)	66.1 ± 10.2	0.648^b^
Yes, Mass casualty event (MCE)	52 (25.5%)	65.4 ± 10.7	
Yes, Natural disaster	4 (2.0%)		
Yes, Military operation	17 (8.3%)		
Yes, War	20 (9.8%)		
Yes, other	30 (14.7%)		
Personal residence near fighting zone			
Yes	23 (11.3%)	67.7 ± 9.7	0.300^c^
Treatment in facility or field under threat			
Yes	115 (56.4%)	65.8 ± 11.5	0.930^b^
Were you or a colleague of yours wounded while treating a patient?			
Yes	24 (11.8%)	66.4 ± 11.0	0.664^b^
A personal loss of a close relative during the current conflict			
Yes	111 (54.4%)	65.4 ± 11.1	0.575^b^
Frequency of stressful events and perceived imminent threat			
Frequency of professional stressful events since Oct. 7th, M ± SD ^**^	4.0 ± 1.8	r = 0.016	0.822^a^
Frequency of personal stressful events since Oct. 7th, M ± SD ^**^	3.8 ± 1.8	r = − 0.083	0.236^a^
Level of personal imminent threat felt during events, M ± SD ^**^	3.2 ± 1.9	r = 0.077	0.272^a^

^a^Based on Pearson correlation; ^b^Based on independent sample t-test; ^c^Based on One-Way ANOVA. ^*^Perceived agency score is represented as M ± SD for categorial variables and as Pearson correlation for continuous variables ^**^Likert scale 1–7; 1-infrequent, 7-very frequent.

#### 3.3.3 Personal characteristics and perceived agency

Resilience demonstrated a positive correlation with perceived agency (r = 0.485, *p* < 0.001) as did the overall psychological capital (PCQ-24) score (r = 0.387, *p* < 0.001). Additionally, significant positive correlations were observed across all four components of the PCQ-24, with correlation coefficients ranging from r = 0.408 for optimism (*p* < 0.001) to r = 0.183 for efficacy (*p* < 0.009). These findings highlight the association between existing psychological resources and perceived agency. Furthermore, we found key statements that are highly indicative of perceived agency, with growth-oriented perspectives showing the highest correlations (Table 4).

**Table 4:** Key statements indicative of high ‘perceived agency’ total score (response to Stressful Experiences Scale).

Key statements of each questionnaire	Correlation with total score of perceived agency*	*p* value
Perceived agency questionnaire		
“During/after medical treatment under significant stress, I find an opportunity for growth.”	r = 0.679	< 0.001
“During/after medical treatment under significant stress, I see it as a challenge that will make me better.”	r = 0.654	< 0.001
“During/after medical treatment under significant stress, I calm and comfort myself.”	r = 0.601	< 0.001
Resilience questionnaire (CD-RISC 10)		
“Coping with stress can make me stronger.”	r = 0.432	< 0.001
“I believe that I can achieve my goals, even if there are obstacles.”	r = 0.395	< 0.001
“I think I am a strong person who can cope with life’s challenges and difficulties.”	r = 0.366	< 0.001
Psychological capital questionnaire (PCQ24)		
“Currently, I see myself as being quite successful at work.”	r = 0.364	< 0.001
“I approach this job as if “every cloud has a silver lining.”	r = 0.363	< 0.001
“I can think of many ways to reach my current work goals.”	r = 0.353	< 0.001

^*^Based on Pearson correlation.

### 3.4 Multivariant analysis and predictors of perceived agency

A linear regression model was employed to evaluate the predictive power of variables associated with perceived agency identified in the univariate analysis (Table 5). The model was statistically significant (*F* = 10.04, degrees of freedom = [11,185], *p* < 0.001), accounting for 37.4% of the variance in perceived agency. Significant predictors as presented in Table 5, include resilience (*β* = 0.342, *p* < 0.001), the hope component of PCQ-24 (*β* = 0.238, *p* = 0.004), education (*β* = −0.156, *p* = 0.035) and religiosity (*β* = 0.142, *p* = 0.022).

**Table 5:** Multivariant analysis - linear regression for predictors of self-efficacy (response to Stressful Experiences Scale).

Variable	Unstandardized Coefficients		Standardized Coefficients		p value
	B	Std. error	β	t	
Constant	20.574	7.229		2.846	0.005
Gender	−0.695	1.400	−0.032	−0.497	0.620
Age	0.113	0.064	0.122	1.773	0.078
Education	−3.641	1.719	−0.156	−2.119	0.035
Religiosity	3.159	1.371	0.142	2.303	0.022
Resilience Levels (CD-RISC 10 score)	0.742	0.168	0.342	4.423	< 0.001
PCQ 24 – Hope component	3.681	1.245	0.238	2.956	0.004
PCQ 24 – Efficacy component	0.206	0.891	0.017	0.231	0.817
PCQ 24 – Resilience component	−2.153	1.343	−0.128	−1.603	0.111
PCQ 24 – Optimism component	1.587	0.918	0.125	1.728	0.086
Profession	2.303	1.652	0.109	1.394	0.165
Treatment environment	0.030	0.704	0.003	0.042	0.967

R^2^ = 0.374, F = 10.04, df _(11,185)._

Variables introduced in Enter mode after negation of multi-collinearity and check for homoscedasticity. Only variables found with a significant association to the dependent variable in the univariant analysis were introduced: Gender (2 categories: Female = 0, Male = 1), Age (continuous), Education (2 categories: Non-Academic = 0, Academic = 1), Religiosity (2 categories: Non-Religious = 0, Religious = 1), Resilience (continuous), PCQ 24 Components (continuous), Profession (2 categories: ‘Doctors and Nurses’ = 0, Paramedics/EMTs = 1), Treatment environment (2 categories: Hospital Environment = 0, Pre Hospital Environment = 1).

## 4 Discussion

This study examined the psychological capital (PsyCap) of HCPs, emphasizing resilience and its effect on perceived agency during MCEs and violent conflict. Findings indicate that hope, personal growth, professional perseverance, spirituality, and emotional regulation are key predictors of coping, exerting stronger influence on resilience and agency than traditional professional factors such as experience and prior exposure. The psychological and societal impacts of the October 7th, 2023, attack on Israel are still unfolding and may parallel outcomes of large-scale natural disasters such as Events like Hurricane Katrina (2005) (22), the 2011 Tōhoku earthquake and tsunami (23), and the Australian bushfires (2019–2020) (24) which caused displacement, destruction, and long-term psychological distress including PTSD. The unprecedented casualties, displacement, and trauma of October 7th will likely pose similar recovery challenges. While early studies have examined mental health consequences among survivors (25–27), our focus was HCPs perceived agency and effectiveness while treating. Positive agency has been shown to protect against PTSD, whereas negative agency may be harmful (28–30).

The diverse sample of HCPs across roles, treatment settings (Table 1) and exposure levels (Table 3) supports generalizability to other professionals working under crisis conditions. Prior research links resilience to reduced stress, burnout, and depression (31), and studies during COVID-19 underscored its role in lowering anxiety and improving capacity to function (32). Higher resilience has also been associated with empathy and attentiveness in HCPs (33), and with superior decision-making and adaptability in military contexts (21). Resilience, a core PsyCap component, enables composure, adaptability, and sound decision-making under stress (34, 35).

Although personality traits and psychological resources influence resilience and agency (2), interventions to enhance resilience have shown mixed results (36). Professional role and treatment setting also shape performance (37): hospitals provide structured workflows and collaboration but may constrain decision-making (38), while pre-hospital settings demand rapid, autonomous action under resource limitations—fostering adaptability yet increasing stress (39). Our findings mirror these distinctions: paramedics and EMTs reported higher agency than physicians and nurses, and pre-hospital environments were associated with higher efficacy than hospital settings.

Evidence on experience is conflicting—some studies link it to better coping (40), while others suggest greater PTSD vulnerability (41). Although intuitively experience should enhance resilience, our study found no association between prior exposure and perceived agency, indicating exposure alone is insufficient. Instead, coping mechanisms, resilience, and psychological resources play a larger role in determining efficacy. Resilience is increasingly seen as a dynamic process (42) shaped by emotional regulation (43), optimism (44), growth-oriented mindset (45), rather than solely by exposure. The Response to Stressful Experiences Scale, developed in military populations and used in the current research, identified meaning-making, active coping, flexibility, spirituality, and self-efficacy as resilience factors (20), which align with our HCP findings.

These results suggest resilience-building interventions across healthcare should extend beyond accumulating experience. Programs may strengthen PsyCap (46), promote adaptive coping (47), and encourage post-traumatic growth (48). Developing internal psychological resources may provide more reliable protection than exposure-based adaptation.

Resilience was strongly associated with perceived agency under pressure. Defined as the ability to “bounce back” or “bounce forward,” it supports crisis response and fosters post-traumatic growth (9). Religiosity, conceptualized as a spiritual dimension of life, correlates positively with mental health and well-being (49, 50),suggesting possible value in integrating spiritual dimensions into HCP training. A 2012 study by Falb and Pargament highlighted mindfulness practices as a potential pathway for developing spiritual qualities (51).

Future research can further examine how environments, exposures, and training affect resilience, and test interventions designed to strengthen it. Additionally, linking perceived efficacy with objective performance measures could yield important insights.

### 4.1 Implication for health policy

Findings of the research carry significant implications for health policy, particularly regarding the preparedness, support, and retention of healthcare personnel during and after large-scale emergencies. Current health policies often emphasize clinical training and operational readiness, yet our results suggest the need to institutionalize psychological preparedness as a core component of professional development. Incorporating resilience-building interventions—such as stress regulation techniques, cognitive reframing, and mindfulness-based practices into training frameworks, could enhance functional capacity under stress. Tailoring these initiatives to specific clinical roles and treatment environments may be important, given significant differences among professional groups and settings (38, 39). Training and support strategies reflect the unique psychological demands faced by hospital-based versus pre-hospital providers. Additionally, the demonstrated association between hope, spirituality, and psychological well-being suggests that spiritual support in various methods, may be a valuable addition to workforce resilience strategies (49, 50, 52). Importantly, the absence of a consistent link between prior exposure and perceived efficacy, challenges assumptions embedded in many emergency preparedness models (40), and points toward the need for proactive psychological resource development. Integrating psychological capital assessments and development opportunities into routine professional evaluations may help standardize and sustain these efforts. By expanding the definition of readiness to include psychological resilience, health systems may better protect and empower their workforce, ensuring more effective, sustainable care delivery in the face of future crises. As global challenges increasingly expose providers to high-intensity stressors, policies that prioritize psychological resources will be essential in safeguarding both workforce stability and population health outcomes, especially in resource-restricted settings.

### 4.2 Limitations

This study has several limitations. Outcomes relied on HCPs’ self-reports, which may not reflect objective functioning. Voluntary participation and non-probabilistic sampling introduce potential selection bias. Data were collected after the initial peak of exposure, but participants may not have fully consolidated their perspectives, raising the possibility of recall bias. A potential limitation of this study is that resilience constitutes a core component of psychological capital (PsyCap), while also being examined as a distinct variable in the analyses. This conceptual overlap may introduce concerns regarding circularity or shared variance, potentially biasing statistical estimates. However, diagnostic testing indicated that multicollinearity was not a significant concern within the analytical models. Finally, the cross-sectional design limits causal inference between resilience and perceived agency.

## 5 Conclusion

Resilience and psychological capital, specifically hope, personal growth, professional perseverance and spirituality were found to be key determinants of HCPs’ perceived agency in crises. Embedding resilience-building into public health systems, alongside organisational and policy measures, may enhance workforce readiness across conflict, disaster, and health threats. These findings are especially relevant to resource-limited settings, where psychological preparedness may be a cost-effective means to support frontline staff and sustain delivery.

## Data Availability

The raw data supporting the conclusions of this article will be made available by the authors, without undue reservation.

## References

[1] Murthy RS Lakshminarayana R. Mental health consequences of war: a brief review of research findings. World Psychiatry. (2006) 5:25–30. 16757987 PMC1472271

[2] Matheson C Robertson HD Elliott AM Iversen L Murchie P. Resilience of primary healthcare professionals working in challenging environments: a focus group study. Br J Gen Pract. (2016) 66:e507–15. doi: 10.3399/bjgp16X685285, 27162205 PMC4917054

[3] Elhadi M Msherghi A Elgzairi M Alhashimi A Bouhuwaish A Biala M . The mental well-being of frontline physicians working in civil wars under coronavirus disease 2019 pandemic conditions. Front Psych. (2020) 11:598720. doi: 10.3389/fpsyt.2020.598720, 33542695 PMC7852461

[4] Kimhi S Eshel Y. Individual and public resilience and coping with long-term outcomes of war1. J Appl Biobehav Res. (2009) 14:70–89. doi: 10.1111/j.1751-9861.2009.00041.x

[5] Duan Y He J Zheng R Feng X Xiao H. The relationship between disaster preparedness, psychological capital, and coping style among nurses: a cross-sectional study from China. Perspect Psychiatr Care. (2022) 58:2577–84. doi: 10.1111/ppc.13097, 35478409

[6] Usta G Bekircan E. Examination of psychological capital and secondary traumatic stress levels of professionals attended to disaster studies. Sociol Spectr. (2024) 44:97–109. doi: 10.1080/02732173.2024.2305963

[7] Fitria D Mustikasari M Panjaitan RU. The psychological capital and anxiety felt by post-market fire disaster victims. J Ners. (2020) 15:1–6. doi: 10.20473/jn.v15i1.17363

[8] Fang S(E) Prayag G Ozanne LK de Vries H. Psychological capital, coping mechanisms and organizational resilience: insights from the 2016 Kaikoura earthquake, New Zealand. Tour Manage Perspect. (2020) 34:100637. doi: 10.1016/j.tmp.2020.100637

[9] Adini B Kimhi S. Perspective: lessons learned from the COVID-19 pandemic concerning the resilience of the population. Isr J Health Policy Res. (2023) 12:19. doi: 10.1186/s13584-023-00557-w, 37131246 PMC10153045

[10] O’Dowd E O’Connor P Lydon S Mongan O Connolly F Diskin C . Stress, coping, and psychological resilience among physicians. BMC Health Serv Res. (2018) 18:730. doi: 10.1186/s12913-018-3541-8, 30241524 PMC6151040

[11] Ataria Y. Sense of ownership and sense of agency during trauma. Phenom Cogn Sci. (2015) 14:199–212. doi: 10.1007/s11097-013-9334-y

[12] Jaffe E Dadon Z Alpert EA. Prehospital care under fire: strategies for evacuating victims from the mega terrorist attack in Israel on October 7, 2023. Prehosp Disaster Med. (2024) 39:275–8. doi: 10.1017/S1049023X24000438, 39291671 PMC11496206

[13] Israel Ministry of Health. (2020). Available online at: https://datadashboard.health.gov.il/portal/dashboard/health (Accessed March 27, 2025).

[14] Fergusson DM Horwood LJ Boden JM Mulder RT. Impact of a major disaster on the mental health of a well-studied cohort. JAMA Psychiatry. (2014) 71:1025–31. doi: 10.1001/jamapsychiatry.2014.652, 25028897

[15] Luthans F Avolio BJ Avey JB Norman SM. Positive psychological capital: measurement and relationship with performance and satisfaction. Pers Psychol. (2007) 60:541–72. doi: 10.1111/j.1744-6570.2007.00083.x

[16] Cid DT Martins M d CF Dias M Fidelis ACF. Psychological capital questionnaire (PCQ-24): preliminary evidence of psychometric validity of the Brazilian version. Psico-USF. (2020) 25:63–74. doi: 10.1590/1413-82712020250106

[17] Connor KM Davidson JRT. Development of a new resilience scale: the Connor-Davidson resilience scale (CD-RISC). Depress Anxiety. (2003) 18:76–82. doi: 10.1002/da.10113, 12964174

[18] Campbell-Sills L Stein MB. Psychometric analysis and refinement of the Connor-Davidson resilience scale (CD-RISC): validation of a 10-item measure of resilience. J Trauma Stress. (2007) 20:1019–28. doi: 10.1002/jts.20271 18157881

[19] Yan X Wang X Xu C Xu Y Liu P Peng L . Psychometric properties of the 10-item Connor-Davidson resilience scale (CD-RISC-10) in male military personnel with and without PTSD. J Affect Disord Rep. (2023) 14:100666. doi: 10.1016/j.jadr.2023.100666

[20] Johnson DC Polusny MA Erbes CR King D King L Litz BT . Development and initial validation of the response to stressful experiences scale. Mil Med. (2011) 176:161–9. doi: 10.7205/milmed-d-10-00258 21366078

[21] Sekel NM Beckner ME Conkright WR LaGoy AD Proessl F Lovalekar M . Military tactical adaptive decision making during simulated military operational stress is influenced by personality, resilience, aerobic fitness, and neurocognitive function. Front Psychol. (2023) 14:1102425. doi: 10.3389/fpsyg.2023.1102425, 36844343 PMC9944034

[22] Rhodes J Chan C Paxson C Rouse CE Waters M Fussell E. The impact of hurricane Katrina on the mental and physical health of low-income parents in New Orleans. Am J Orthopsychiatry. (2010) 80:237–47. doi: 10.1111/j.1939-0025.2010.01027.x, 20553517 PMC3276074

[23] Nicole W. Aftershocks: home loss and long-term health after the 2011 great East Japan earthquake. Environ Health Perspect. (2022) 130:84002. doi: 10.1289/EHP11792, 35960037 PMC9373854

[24] Zhang Y Workman A Russell MA Williamson M Pan H Reifels L. The long-term impact of bushfires on the mental health of Australians: a systematic review and meta-analysis. Eur J Psychotraumatol. (2022) 13:2087980. doi: 10.1080/20008198.2022.2087980, 35957633 PMC9359172

[25] Mayer Y Shiffman N Bergmann E Natoor M Khazen S Lurie I . Mental health outcomes of Arab and Jewish populations in Israel a month after the mass trauma events of October 7, 2023: a cross-sectional survey of a representative sample. Psychiatry Res. (2024) 339:116042. doi: 10.1016/j.psychres.2024.116042, 38945101

[26] Levi-Belz Y Groweiss Y Blank C Neria Y. PTSD, depression, and anxiety after the October 7, 2023 attack in Israel: a nationwide prospective study. EClinicalMedicine. (2024) 68:102418. doi: 10.1016/j.eclinm.2023.102418, 38586476 PMC10994954

[27] Elyoseph Z Hadar-Shoval D Angert T Yitshaki N Hol E Asman O . Mental health volunteers after the Oct 7 Gaza border crisis in Israel: silent warriors. Lancet Psychiatry. (2024) 11:10–2. doi: 10.1016/S2215-0366(23)00369-3, 37952553

[28] Schwaab L. Why we need to consider agency to understand traumatization: a critical psychological approach. Annual Rev Critical Psychol. (2024) 18:508–20.

[29] Huang H Kashubeck-West S. Exposure, agency, perceived threat, and guilt as predictors of posttraumatic stress disorder in veterans. J Counsel Dev. (2015) 93:3–13. doi: 10.1002/j.1556-6676.2015.00176.x

[30] Tapal A Oren E Dar R Eitam B. The sense of agency scale: a measure of consciously perceived control over one’s mind, body, and the immediate environment. Front Psychol. (2017) 8:1552. doi: 10.3389/fpsyg.2017.01552 28955273 PMC5600914

[31] Simpkin AL Khan A West DC Garcia BM Sectish TC Spector ND . Stress from uncertainty and resilience among depressed and burned out residents: a cross-sectional study. Acad Pediatr. (2018) 18:698–704. doi: 10.1016/j.acap.2018.03.002, 29524616

[32] Huffman EM Athanasiadis DI Anton NE Haskett LA Doster DL Stefanidis D . How resilient is your team? Exploring healthcare providers’ well-being during the COVID-19 pandemic. Am J Surg. (2021) 221:277–84. doi: 10.1016/j.amjsurg.2020.09.005, 32994041 PMC7486626

[33] Aljarboa BE Pasay An E Dator WLT Alshammari SA Mostoles R Uy MM . Resilience and emotional intelligence of staff nurses during the COVID-19 pandemic. Healthcare (Basel). (2022) 10. doi: 10.3390/healthcare10112120 PMC9691039 36360460

[34] Kovacs I Irimia A Pupazan D Ilie C Gireada A. Research on resilience of intervention and rescue personnell. 23rd international multidisciplinary scientific GeoConference proceedings SGEM 2023, energy and clean technologies, Vol 23. STEF92 Technology; (2023). p. 117–124. doi: 10.5593/sgem2023/4.1/s17.15

[35] Karrasch S Hitzler M Behnke A Tumani V Kolassa I-T Rojas R. Chronic and traumatic stress among emergency medical services personnel. Z Klin Psychol Psychother. (2020) 49:204–17. doi: 10.1026/1616-3443/a000600

[36] Venegas CL Nkangu MN Duffy MC Fergusson DA Spilg EG. Interventions to improve resilience in physicians who have completed training: a systematic review. PLoS One. (2019) 14:e0210512. doi: 10.1371/journal.pone.0210512, 30653550 PMC6336384

[37] O’Rourke MW White A. Professional role clarity and competency in health care staffing--the missing pieces. Nurs Econ. (2011) 29:183–8. 21919415

[38] Brennan MD Monson V. Professionalism: good for patients and health care organizations. Mayo Clin Proc. (2014) 89:644–52. doi: 10.1016/j.mayocp.2014.01.011, 24797645

[39] Lord B. Book review: “clinical reasoning in the health professions” “clinical reasoning in the health professions” edited by Higgs and JonesJ. M. 2000, 2ndedition, Butterworth-Heinemann, Oxford. 336 pages, ISBN 0-750-63907-5 recommended retail price: $134.75.AUD. Australas J Paramed. (2003) 1:1–2. doi: 10.33151/ajp.1.3.193

[40] Hytten K Hasle A. Fire fighters: a study of stress and coping. Acta Psychiatr Scand Suppl. (1989) 355:50–5. doi: 10.1111/j.1600-0447.1989.tb05253.x 2624134

[41] Fullerton CS Ursano RJ Wang L. Acute stress disorder, posttraumatic stress disorder, and depression in disaster or rescue workers. Am J Psychiatry. (2004) 161:1370–6. doi: 10.1176/appi.ajp.161.8.1370 15285961

[42] Southwick SM Bonanno GA Masten AS Panter-Brick C Yehuda R. Resilience definitions, theory, and challenges: interdisciplinary perspectives. Eur J Psychotraumatol. (2014) 5. doi: 10.3402/ejpt.v5.25338 PMC4185134 25317257

[43] Rao GP Koneru A Nebhineni N Mishra KK. Developing resilience and harnessing emotional intelligence. Indian J Psychiatry. (2024) 66:S255–61. doi: 10.4103/indianjpsychiatry.indianjpsychiatry_601_23 38445274 PMC10911335

[44] Tugade MM Fredrickson BL. Resilient individuals use positive emotions to bounce back from negative emotional experiences. J Pers Soc Psychol. (2004) 86:320–33. doi: 10.1037/0022-3514.86.2.320 14769087 PMC3132556

[45] Tao W Zhao D Yue H Horton I Tian X Xu Z . The influence of growth mindset on the mental health and life events of college students. Front Psychol. (2022) 13:821206. doi: 10.3389/fpsyg.2022.821206, 35496212 PMC9046553

[46] Vîrgă D Baciu E-L Lazăr T-A Lupșa D. Psychological capital protects social workers from burnout and secondary traumatic stress. Sustainability. (2020) 12:2246. doi: 10.3390/su12062246

[47] Miller O Shakespeare-Finch J Bruenig D. Predicting burnout, well-being, and posttraumatic growth in correctional officers. Crim Justice Behav. (2024) 51:724–42. doi: 10.1177/00938548241233932

[48] Xu X Hu M-L Song Y Lu Z-X Chen Y-Q Wu D-X . Effect of positive psychological intervention on posttraumatic growth among primary healthcare workers in China: a preliminary prospective study. Sci Rep. (2016) 6:39189. doi: 10.1038/srep39189, 27995960 PMC5171914

[49] Rawdin B Evans C Rabow MW. The relationships among hope, pain, psychological distress, and spiritual well-being in oncology outpatients. J Palliat Med. (2013) 16:167–72. doi: 10.1089/jpm.2012.0223, 23101471 PMC3569921

[50] Fehring RJ Miller JF Shaw C. Spiritual well-being, religiosity, hope, depression, and other mood states in elderly people coping with cancer. Oncol Nurs Forum. (1997) 24:663–71. 9159782

[51] Falb MD Pargament KI. Relational mindfulness, spirituality, and the therapeutic bond. Asian J Psychiatr. (2012) 5:351–4. doi: 10.1016/j.ajp.2012.07.008, 23174445

[52] Powell JL. "Religiosity and spirituality" In: Carducci BJ Nave CS Nave CS, editors. The Wiley encyclopedia of personality and individual differences: Models and theories. Hoboken, New Jersey, USA: Wiley (2020). 417–21. doi: 10.1002/9781118970843.ch334

